# Meausures of organizational characteristics associated with adoption and/or implementation of innovations: A systematic review

**DOI:** 10.1186/s12913-017-2459-x

**Published:** 2017-08-23

**Authors:** Jennifer D. Allen, Samuel D. Towne, Annette E. Maxwell, Lisa DiMartino, Bryan Leyva, Deborah J Bowen, Laura Linnan, Bryan J. Weiner

**Affiliations:** 1 Department of Community Health, Tufts University, 574 Boston Avenue, Medford, MA 02155 USA; 2 Texas A&M University, School of Public Health, 1266 TAMU, College Station, TX 77843-1266 USA; 3 University of California Los Angeles, Fielding School of Public Health, 650 Charles Young Drive South, Los Angeles, CA 90095-6900 USA; 4 Department of Health Policy and Management CB#7411, University of North Carolina at Chapel Hill, Chapel Hill, NC 27514-7411 USA; 5 Warren Alpert Medical School, Brown University, Providence, RI 02903 USA; 6 Department of Bioethics and Humanities, University of Washington, 1107 ne 45th street #305, Seattle, WA 98105 USA; 7 Department of Health Behavior, University of North Carolina Gillings School of Global Public Health, University of North Carolina at Chapel Hill, CB 7440, Chapel Hill, NC 27599-7440 USA; 8 Department of Health Policy and Management, University of North Carolina Gillings School of Public Health, University of North Carolina at Chapel Hill, CB #7411, Chapel Hill, NC 27599-7400 USA

**Keywords:** Systematic review, Organizational characteristics, Measures, Innovation, Adoption and implementation, Innovations, Consolidated framework for implementation research

## Abstract

**Background:**

This paper identifies and describes measures of constructs relevant to the adoption or implementation of innovations (i.e., new policies, programs or practices) at the organizational-level. This work is intended to advance the field of dissemination and implementation research by aiding scientists in the identification of existing measures and highlighting methodological issues that require additional attention.

**Methods:**

We searched for published studies (1973–2013) in 11 bibliographic databases for quantitative, empirical studies that presented outcome data related to adoption and/or implementation of an innovation. Included studies had to assess latent constructs related to the “inner setting” of the organization, as defined by the Consolidated Framework for Implementation Research.

**Results:**

Of the 76 studies included, most (86%) were cross sectional and nearly half (49%) were conducted in health care settings. Nearly half (46%) involved implementation of evidence-based or “best practice” strategies; roughly a quarter (26%) examined use of new technologies. Primary outcomes most often assessed were innovation implementation (57%) and adoption (34%); while 4% of included studies assessed both outcomes. There was wide variability in conceptual and operational definitions of organizational constructs. The two most frequently assessed constructs included “organizational climate” and “readiness for implementation.” More than half (55%) of the studies did not articulate an organizational theory or conceptual framework guiding the inquiry; about a third (34%) referenced Diffusion of Innovations theory. Overall, only 46% of articles reported psychometric properties of measures assessing latent organizational characteristics. Of these, 94% (33/35) described reliability and 71% (25/35) reported on validity.

**Conclusions:**

The lack of clarity associated with construct definitions, inconsistent use of theory, absence of standardized reporting criteria for implementation research, and the fact that few measures have demonstrated reliability or validity were among the limitations highlighted in our review. Given these findings, we recommend that increased attention be devoted toward the development or refinement of measures using common psychometric standards. In addition, there is a need for measure development and testing across diverse settings, among diverse population samples, and for a variety of types of innovations.

**Electronic supplementary material:**

The online version of this article (doi:10.1186/s12913-017-2459-x) contains supplementary material, which is available to authorized users.

## Background

The gap between the generation of new evidence and translation of this evidence into practice is the single biggest chasm in biomedical and community-based research today [1, 2]. The lag between discovery and implementation is partly due to a failure to understand organizational factors that affect adoption and implementation of innovations (i.e., new policies, programs or practices) [3]. Adoption has been defined as the decision of an organization or a community to commit to and initiate an evidence-based intervention (EBI), whereas implementation involves the process of putting to use or integrating an EBIs within a setting [4]. Successful dissemination of EBIs requires an understanding of *how* and *why* organizations and systems adopt innovations, as well as their capacity to deliver and maintain them over time.

While there is a growing literature on organizational characteristics related to adoption and implementation of EBIs, the field is still in need of validated conceptual frameworks and measures. There are important lessons to be learned from other reviews [3, 4] and other disciplines (e.g., education, community psychology, health services) [5–12]. However, to our knowledge, a systematic review of organizational characteristics and methods of measurement across disciplines and settings (e.g., health care, technology firms, schools) has not yet been conducted. To move the field of dissemination and implementation (D&I) in public health forward, there is a need for reliable and valid measures to assess organizational characteristics and to validate conceptual frameworks that seek to guide strategies to promote adoption and implementation of EBI strategies broadly.

Given the early stage of research in the fields of health services and public health, coupled with the rapid pace of advances in science, this is an opportune time to evaluate measures of organizational characteristics and to consider next steps to strengthen them. Thus, the aims of this systematic review are to: (1) identify measures of organizational characteristics hypothesized to be associated with the adoption and/or implementation of innovations across a range of disciplinary fields; (2) describe the characteristics and psychometric properties of the measures; and (3) provide recommendations to improve the measurement of constructs in future studies. While the terms organizational “predictors,” “factors,” and “measures” are often used interchangeably, we chose the term organizational “construct” to refer to the characteristic being evaluated [13]. In this review, we focus specifically on latent constructs [14], or those that cannot be directly observed, and examine the psychometric properties of the variables used to measure those constructs.

## Methods

### Conceptual model

We used the Consolidated Framework for Implementation Research (CFIR) to guide the entries in this systematic review [15]. The CFIR is a meta-theoretical framework that integrates constructs from relevant D&I theories [16] and addresses multiple domains, including the: (1) inner organizational setting; (2) characteristics of the intervention or EBI; (3) outer setting; (4) characteristics of those implementing the intervention; and (5) processes put in place for implementation. The CFIR is becoming a widely used framework in D&I research [15, 17], and thus provided a useful framework for the current review.

Our main goal was to identify and review measures of organizational characteristics associated with adoption and/or implementation of innovations. Our review focused on latent constructs associated with CFIR’s “inner organizational setting” because we were particularly interested in describing psychometric properties of existing measures. Thus, we excluded/omitted descriptions of structural features of organizations that are directly observable, such as organizational size. The latent constructs described under the inner organizational setting are comprised of: (a) networks and communications (e.g., teamwork, relationships among individuals within the organization); (b) culture (e.g., norms, values); and (c) implementation climate, which is described as “shared receptivity to change.” Receptivity includes six sub-constructs, including tension for change, compatibility of change with organizational values, relative priority (shared perception of importance of change), organizational incentives and rewards for change goals and feedback (“extent to which goals are clearly communicated, acted upon”), learning climate, and readiness for implementation [15]. According to the CFIR, an inner organizational setting that is stable, has clear and decentralized decision-making authority, has a capacity for change, and collective receptivity to change is more likely to adopt and/or implement an innovation than those lacking these characteristics [15].

### Identification of eligible articles

With assistance from a Health Services Reference Librarian, we generated a comprehensive list of search terms and used a database thesauri to tailor the list of terms for each of 11 relevant electronic databases. Databases searched included: PubMed (Web-based), EMBASE (DIALOG platform), Cumulative Index to Nursing and Allied Health Literature (CINAHL—EBSCO platform), ABI/INFORM (DIALOG), Business Source Premier (EBSCO), PsycINFO (DIALOG), EconLit (DIALOG), Social Sciences Abstracts (DIALOG), SPORTDiscus (EBSCO), ILLUMINA and Educational Resource Information Center (ERIC). In PubMed, the following Medical Subject Headings (MeSH) terms were selected and combined with relevant title and text words: *organizational culture*, *organizational objectives*, *leadership*, *organizational policy*, *organizational innovation*, *diffusion of innovations*, and *efficiency—organizational.* Title and text words defined the concepts of *organizational characteristics*, *organizational factors*, *organizational capacity*, *measures*, *adoption*, *implementation*, *sustainability*, *decision-making,* and *readiness*. Although papers offered various definitions for adoption and implementation specific to the innovation studied, adoption was generally conceptualized as the decision process of an organization to commit to and utilize an innovation. Implementation was conceptualized as putting to use or integrating an innovation within a setting. Search strategies for the remaining databases were adjusted for the syntax appropriate for each database using a combination of thesauri and text words. Database searches were conducted between January and March of 2013. Papers included had to: (a) be written in the English language; (b) be published in a peer-reviewed journal; (c) report results of original research; (d) quantitatively assess outcomes related to adoption and/or implementation of innovations; and (e) assess latent constructs of the “inner organizational setting” as defined by the CFIR. We chose 1973 as a start date for the review because we wanted to include contributions to the field of D&I from other disciplines and this is when research on D&I seemed to become very active. All published reports from January 1973 to March 2013 were identified and retrieved.

### Data abstraction and coding

Information regarding several key variables was abstracted. The complete list of variables included: year of publication, study location (country), study design, year of data collection (i.e., reported within the study), sample characteristics, study setting (e.g., health care, worksite), type of innovation (e.g., evidence-based/best practice strategies), outcomes (i.e., adoption, implementation, implementation and adoption, organizational readiness), use of theory, name of theory (e.g., Diffusion of Innovations), constructs assessed, inclusion/exclusion of psychometric properties (i.e., reliability, validity), and characteristics of measures used to assess each construct. Assessment of study quality (i.e., potential bias) was not conducted in the process of this review. Our main goal was to describe the psychometric properties of the widest possible range of available measurement instruments; studies of poor quality may or may not include good organizational measures.

To ensure consistency of coding, a standardized codebook was created based on a prior measurement review [18]. As a next step, all research team members reviewed three of the included studies and abstracted relevant data. Team members then reviewed the classification of measures for each of the constructs in each of the papers and discussed any disagreements until consensus was reached. The codebook was thereafter refined. During the subsequent coding process, three research team members independently reviewed full texts using pre-determined inclusion and exclusion criteria. Three research team members coded all papers independently; one additional team member subsequently verified all coding. Data were subsequently added to an Access database; after cleaning, the data were downloaded into Excel files and evidence tables were constructed, and organized by CFIR inner organizational construct categories. All excluded papers underwent full-text review by at least three authors to verify exclusion criteria.

Discrepancies in coding within triads were resolved through discussion with the research team. We opted for a group consensus method because of the large number of papers and the enormous variability in terminology used to describe organizational characteristics across studies. We elected to follow standard definitions of organizational constructs provided by the CFIR [12], as opposed to the terminology selected by the authors, due to this wide variability. We used the PRISMA guidelines [19] to report the process and results of this review.

## Results

### Citations included

**Fig. 1:**
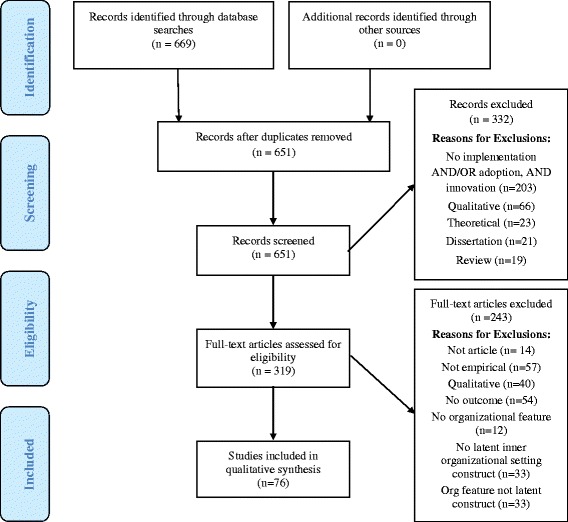
PRISMA flow diagram

The yield from each phase of the search process is depicted in Fig. 1. After duplicates were removed, a total of *n* = 651 unique citations were identified and reviewed for eligibility as described. A total of *n* = 332 (51%) articles were excluded during the initial review process. Of the *n* = 319 (49%) articles that underwent full-text review, *n* = 243 (76%) were ultimately excluded because they did not meet one or more of the inclusion criteria. Nearly half of exclusions (46%) were due to the article type (e.g. not peer-reviewed article); 22% of excluded studies lacked outcome data. This multi-stage review process yielded *n* = 76 articles for inclusion in the review (Table 1).

**Table 1:** Characteristics of included studies (*n* = 76 studies)

	n	%
Study design		
Cross-sectional	66	86
Non-randomized trial (cluster)	5	7
Other	5	7
Study location		
United States	48	63
Canada	7	9
Other	21	28
Data collection method		
Mail survey	44	59
Online/email	7	9
Interviewer-administered survey	8	10
Mixed methods	8	10
Other/not specified	9	12
Study setting		
Health care	37	49
Worksite	17	22
School	12	16
Community/public health/social services	10	13
Sample (respondent category)		
Employees	31	41
Executives	13	17
Principal/teachers	12	16
Nurses/medical doctors/clinical staff	14	18
Other/missing	6	8
Primary outcome		
Adoption	26	34
Implementation	43	57
Implementation & adoption	3	4
Organizational readiness	4	5
Type of innovation		
Evidence-based/best practice strategies	35	46
Administrative	13	17
Technological	20	26
Administrative & technological	5	7
Combination (< 1 innovation type)	2	3
Not specified	1	1
Theories of organizational change		
Diffusion of Innovations	26	34
Other	8	11
None cited	42	55

### Characteristics of included studies

Table 2 shows the characteristics of the studies included in the review. A majority (63%) took place in the U.S. Most (86%) were cross-sectional and over half used mailed surveys for data collection (59%). Nearly half (49%) of studies were conducted in health settings, which included physical and mental health service agencies. Studies in worksites (22%) and schools (16%) were also relatively common. About half (46%) of the “innovations” that were studied included evidence-based strategies or ‘best practices,’ although there were a substantial number (26%) that addressed technological innovations.

**Table 2:** CFIR Inner Setting Constructs and Definitions [15]

Construct	Definition
Structural Characteristics	The social architecture, age, maturity, and size of an organization.
Networks & Communications	The nature and quality of webs of social networks and the nature and quality of formal and informal communications within an organization.
Organizational Culture	Norms, values, and basic assumptions of a given organization.
Implementation Climate	The absorptive capacity for change, shared receptivity of involved individuals to an intervention and the extent to which use of that intervention will be rewarded, supported, and expected within their organization.
Learning climate	A climate in which: a) leaders express their own fallibility and need for team members’ assistance and input; b) team members feel that they are essential, valued, and knowledgeable partners in the change process; c) individuals feel psychologically safe to try new methods; and d) there is sufficient time and space for reflective thinking and evaluation.
Compatibility	The degree of tangible fit between meaning and values attached to the intervention by involved individuals, how those align with individuals’ own norms, values, and perceived risks and needs, and how the intervention fits with existing workflows and systems.
Goals and Feedback	The degree to which goals are clearly communicated, acted upon, and fed back to staff and alignment of that feedback with goals.
Relative Priority	Individuals’ shared perception of the importance of the implementation within the organization.
Organizational Incentives & Rewards	Extrinsic incentives such as goal-sharing awards, performance reviews, promotions, and raises in salary and less tangible incentives such as increased stature or respect.
Tension for Change	The degree to which stakeholders perceive the current situation as intolerable or needing change.
Readiness for Implementation	Tangible and immediate indicators of organizational commitment to its decision to implement an intervention.
Leadership Engagement	Commitment, involvement, and accountability of leaders and managers with the implementation.
Available Resources	The level of resources dedicated for implementation and on-going operations including money, training, education, physical space, and time.
Access to Knowledge and Information	Ease of access to digestible information and knowledge about the intervention and how to incorporate it into work tasks.

More of the literature focused on implementation (57%) than adoption (34%), with few (4%) addressing both. Nearly half (45%) of the studies specifically cited a theory or conceptual model guiding the investigation; of those that did, the largest percentage (34%) cited Diffusion of Innovations Theory.

### Psychometric properties of measures

Details of each of the studies are included in Additional file 1. Overall, of the 76 included studies, approximately 46% (*n* = 35) included psychometric information pertaining to organizational measures. Because many studies assessed more than one inner organizational construct measure, the number of studies included in the table (*n* = 35) is less than the total number of constructs assessed (*n* = 83). Of the total number of studies that included some psychometric information about measures, 94% (33/35) described reliability and 71% (25/35) reported on validity.

Among those studies with reliability reported (*n* = 33), most reported internal consistency reliability with Cronbach’s alphas (85%, *n* = 28/33). One study did not report actual measures of reliability or validity directly in the manuscript, but did provide a citation referencing reliability and validity. Among those studies with validity reported (*n* = 25) the most common forms of validity included content, discriminant, and convergent validity. Information on potential biases within and/or across studies was not specified in the current study, but addressed more broadly in the discussion.

There were a total of 83 measures of CFIR constructs identified. Across all of the studies included in this review, only one measure was used in more than one study [20]. Across studies, definitions of constructs varied widely. For example, “structural characteristics” included, but were not limited to, organizational structure, formalization, and centralization. The two most frequently reported organizational constructs across the studies were “readiness for implementation” (60%, *n* = 21/35) and “organizational climate” (54%, *n* = 19/35). The number of items used to assess each construct ranged from one to 32, with the majority of measures consisting of three to six items.

## Discussion

While a few reports on measurement in the field of D&I research have been published [3, 21, 22], we believe this systematic review adds to the literature in that it was guided by an explicit and widely-used conceptual model (i.e., CFIR), included studies of both adoption and implementation, integrated literature from multiple disciplines, and considered a variety of settings and innovation types. This review confirms the lack of standardized or validated instruments to assess organizational characteristics.

### Inconsistent definitions of constructs and lack of information on reliability and validity for organizational measures

Our findings are similar to others that have examined the issue of measurement in D&I studies [3, 8] and have found inconsistent definitions of constructs and lack of information on reliability and validity for organizational constructs. For example, Wisdom and colleagues [23] studied factors influencing adoption of innovations across multiple theoretical frameworks and in multiple settings (e.g., health care, government bodies), noting that implementation cannot occur without adoption [23]. While organizational-level measures were among the most likely to be assessed in the adoption process, the authors found no consistency in measures across studies in their review. Martinez and colleagues [24] reviewed instrumentation challenges facing the field of implementation science and recommended that investigators utilize the Society for Implementation Research Collaboration (SIRC) Instrument Review Project and the Grid-Enabled Measures (GEM) databases to identify existing instruments [21]. We agree with this recommendation but note that there remains a need for additional research and development on organizational level measures. Kruse and colleagues [25] conducted a systematic review of factors influencing adoption of the electronic health record in medical practice and produced a conceptual model of internal and external organizational characteristics factors that they believe should guide future empirical tests [12]. However, their review combined a wide array of internal and external factors; differences between organizational and individual or interpersonal factors that might influence the adoption decision were not clearly delineated. More recently, Clinton-McHarg and colleagues [22] identified gaps in reporting of psychometric properties of organizational measures and concluded that such omissions limit the utility of implementation-focused theoretical frameworks. A review of measures by Kirk and colleagues [21] found increasing use of the CFIR in D&I studies, but advocate for explicit justifcation for inclusion/exclusion of particular constructs within the framework. Each of the above-mentioned reviews provide important contributions to the field and demonstrate a broad consensus regarding the need for improved scrutiny and development of organizational level measures.

Relative to other fields of study, D&I research in public health and health care is a relatively new priority [9]. As such, it is not surprising that there is a lack of standardized or validated measures to assess salient organizational characteristics. However, increased attention to the development of measures of organizational characteristics is required to improve our understanding of important and potentially modifiable influences on adoption and/or implementation of health program or service-related innovations. Given the few standardized or validated measures identified in this review, we emphasize the need for the development of measures using common psychometric standards [24, 26]. To advance the field, it is crucial that all measures of latent constructs undergo rigorous evaluation of reliability and validity across diverse settings, among diverse population samples, and for a variety of types of innovations.

### NIH-funded grid-enabled measures (GEM) database

A positive step in the direction toward improved measures is the NIH-funded Grid-Enabled Measures (GEM) database – a web-based tool to collect and create a national database of measures that can enhance the current state of the science on organizational measures. GEM contains behavioral, social science, and other relevant scientific measures organized by theoretical constructs. The website also contains ‘workspaces’ related to specific scientific disciplines to promote collaboration among researchers while building consensus regarding the use of quality measures. The GEM-D&I workspace currently contains over 130 measures, however, organizational level constructs have received relatively little attention to date [19]. The Instrument Review Project (IRP) led by the Society for Implementation Research Collaboration (SIRC) [26] is an exciting new effort that seeks to address this gap on organizational level measures in mental health, health care and school settings. In addition to providing reviews of existing D&I methods, the SIRC IRP provides a synthesis of implementation science instruments and measures, citations to sources of the measurements, as well as the actual measures [20]. Having a central repository of instruments will allow D&I researchers to obtain and use measures more thoughtfully, enable cross-comparison of study findings, and facilitate the sharing of harmonized data.

### Theoretical models or frameworks

Another way to enhance the likelihood of successful adoption and implementation is to address an important limitation we found in our review – a lack of studies that use conceptual models or frameworks to guide the research. This problem has been cited as a barrier to theory-based inquiry [9], but the good news is that new frameworks have been gaining traction in recent years [12, 15, 27, 28] and can provide the basis for greater conceptual clarity in terms of construct definitions, designation of variables as mediators or moderators, and can assist with the operationalization of measures.

This review found that Rogers’s Diffusion of Innovation [16] was the most commonly cited theory. Diffusion of Innovations [16] has been generalized across clinical health care settings [29–31] and applied in other industries (e.g., steel firms [32], home building [33]). Thus, it is likely that organizational measures related to Diffusion of Innovations applied in one setting could be developed and/or adapted for use across different industry sectors, types of innovations and populations and cultural groups [34]. More theory-guided research that incorporates organizational-level measures will enhance both research and practice.

### Recommendations for future research

Based on these results, our team summarized several important recommendations to be considered in future research. First, we advocate for the use of *standardized reporting guidelines*, such as for clinical trials (CONSORT) [35], non-randomized designs (TREND) [36], or STROBE (for observational studies) [37] and perhaps adapting these guidelines to include specific information relevant to D&I studies. Such detail could help to ensure the availability of information required to evaluate the strengths and limitations of research. The inclusion of additional detail about the development or adaptation of existing measures, as well as strategies to assess validity and reliability, would facilitate methodological and programmatic advancements. Very recently, Neta et al. [38] proposed a framework for enhancing the value of D&I research which emphasized the importance of considering better reporting at several steps along the research process: planning, delivery, evaluation/results reporting and long-term outcomes. To optimize the value of D&I research, they advocate for distinct reporting guidelines at each of these steps, along with relevant measures. Yet, they acknowledge that “the major barrier to reporting of these measures is the lack of practical, validated, well-accepted instruments (and analytic approaches)” [38].

Our team also recommends that more *mixed methods research*, where both qualitative and quantitative methodologies are brought to bear on the understanding of D&I issues, is desirable. Our understanding of implementation is rapidly evolving and we are not yet sure which theoretical models are most appropriate and/or applicable to certain situations, nor are we clear about the best definitions of all constructs that are included in theoretical models or conceptual frameworks. Qualitative research methods provide an excellent way to conduct exploratory work that will yield important insights for designing new measures and identifying salient factors that might not currently be reflected in existing conceptual models. Indeed, qualitative investigations of organizational characteristics associated with adoption or implementation [39–52] have greatly enhanced our understanding of relevant factors and helped to refine existing theories. By taking advantage of the strengths of both qualitative and quantitative methods, it is possible to strengthen measure design, development and testing as well.

Results of this review revealed that *organizational characteristics can be conceptualized and measured at multiple levels*. We recommend that researchers specify and assure congruence among the level of theory, the level of measurement, and the level of analysis [53–55]. Culture, climate, leadership, power, participation, and communication are constructs of potential importance to adoption and implementation that can be conceptualized at the organizational level of theory, even though the source of data for the construct resides at the level of an individual [13]. When individuals are asked to report their perceptions of organizational characteristics, it will be important to employ methods to reduce the potential for social desirability bias. This can be accomplished by utilizing neutral question wording, emphasizing the need for respondents to focus on organizational characteristics as opposed to their individual opinions or attitudes, and/or data collection from multiple individuals within a single organization. In working with these organizational constructs, we also recommend that researchers specify the composition model that links the lower-level data to the higher-level construct [53–57]. Several composition models exist (e.g., direct consensus, referent shift, dispersion, and frog-pond) and researchers can use several statistical measures to verify that the functional relationship specified in the composition model holds for the data in question [54]. Specification of the composition model and assessment of its fit to the data increase confidence in the reliability and validity of higher-level constructs measured with lower-level data.

#### Limitations

Some may view our use of the CFIR as an organizing framework for this systematic review as a limitation of the research. While the CFIR is a widely accepted meta-theoretical framework, it is only one of a growing number of theories, models, and frameworks. Theoretical models and conceptual frameworks to guide D&I inquiries are receiving increased attention [58]. On the other hand, we are unaware of any other theory-guided reviews and believe that this is a major strength of our study. In a recent review, Tabak and colleagues identified more than 61 theoretical models and conceptual frameworks used in implementation science [28] and the number is still growing, as noted in a recent webinar from the Agency for Healthcare Research and Quality (AHRQ) [59]. Nevertheless, even when a single construct that cuts across multiple theories and models is studied (e.g., organizational readiness), there is no consensus regarding conceptual or operational definitions [60].

Another limitation of our review is that relatively little is known about how characteristics of the CFIR “inner organizational setting” relate to outcomes of adoption and implementation [21]. All studies included in this review had an explicit outcome related to adoption and/or implementation, but the extent of adoption or degree of implementation was not consistently identified. And, the vast majority of studies were cross-sectional in nature, not empirical. Thus, we have limited comparison across many factors (level, completeness, frequency, intensity and duration of use) [9]. There is clear need for more studies that examine other domains of the CFIR and test the relationship between key organizational measures and selected adoption or implementation outcomes. Finally, publication bias may have affected the results presented in the current study given that one of our inclusion criteria was being published in a peer-reviewed journal.

## Conclusions

The lack of clarity in construct definitions, inconsistent use of theory, absence of standardized reporting criteria for D&I research, and the fact that few measures have demonstrated reliability or validity were among the limitations highlighted in our review. Given these findings, we recommend that increased attention be devoted toward the development or refinement of measures using common psychometric standards. In addition, there is a need for measure development and testing across diverse settings, among diverse population samples, and for a variety of types of innovations.

## Additional file

Additional file 1: Characteristics of studies (*n*=35) and CFIR constructs (*n*=83) for which psychometric information was reported. (DOCX 104 kb)
