# Supplementary material for: Meausures of organizational characteristics associated with adoption and/or implementation of innovations: A systematic review
Source: BMC Health Serv Res. 2017 Aug 23;17:591. doi: 10.1186/s12913-017-2459-x (PMC5569532; doi:10.1186/s12913-017-2459-x)
Supplement: Additional file 1: — Characteristics of studies (n=35) and CFIR constructs (n=83) for which psychometric information was reported. (DOCX 104 kb) [file 12913_2017_2459_MOESM1_ESM.docx]

**ADDITIONAL FILE**

**Characteristics of studies (n=35) and CFIR constructs (n=83) for which psychometric information was reported**

| **Study (Ref)** | **Measure name** | **Definition** | **Reliability** | **Validity** | **Setting** | **Level of measurement** | **Number of items** |
| --- | --- | --- | --- | --- | --- | --- | --- |
| **Construct: Structural characteristics (5 studies, 12 measures)** | | | | | | | |
| Cooke, 2000  [1] | Centralization | The degree to which decisions about resources and policy are centralized | Cronbach’s alpha  > 0.70  Cited Cooke, Mattick, and Campbell, 1998 [2] | Not reported | Health care | Organization | 5 |
| Cooke, 2000  [1] | Hierarchy | The degree to which decisions about task performance are centralized | Cronbach’s alpha  > 0.70 | Not reported | Health care | Organization | 6 |
| Cooke, 2000  [1] | Formalization | The level of use of rules, regulations and standardization of procedures | Cronbach’s alpha  > 0.70 | Not reported | Health care | Organization | 15 |
| Nieboer, Pijpers, and Strating, 2011[3] | Structure | A clear division in tasks and responsibilities | Cronbach’s alpha = 0.68 | Not reported | Health care | Organization | 10 |
| Nieboer, Pijpers et al., 2011[3] | System | Formal and informal rules, procedures, and information systems | Cronbach's alpha = 0.66 | Not reported | Health care | Organization | 5 |
| Nieboer, Pijpers et al., 2011[3] | Staff | Employee morale and use of tools to help communicate what is important | Cronbach’s alpha = 0.51 | Not reported | Health care | Organization | 5 |
| Nieboer and Strating, 2012[4] | Centralization | Item examples included “little action can be taken here until a supervisor approves the decision”; “unit members need to ask their supervisors before they do almost anything” | Cronbach's alpha = 0.41 | Not reported | Health care | Multilevel | 5 |
| Okafor and Thomas, 2008 [5] | Formalization | Degree to which the organization stresses that its members follow set rules and procedures | Cronbach’s alpha = 0.99 | Construct (content) – expert review  Construct (structural) – confirmatory factor analysis  Construct (convergent and discriminant) – role ambiguity positively associated with formalization  Criterion (predictive) – regression – formalization positively associated with innovation adoption-enabling elements | Health care | Multilevel | 4 |
| Okafor and Thomas, 2008 [5] | Centralization | Extent to which decisions are made by a small nucleus of administrative personnel | Cronbach’s alpha = 0.94 | Construct (content) – expert review  Construct (structural) – confirmatory factor analysis  Construct (convergent and discriminant) – hierarchy of authority negatively correlated with participation in decision making  Criterion (predictive) – regression – participation in decision making positively associated with innovation adoption-enabling elements | Health care | Multilevel | 5 |
| Okafor and Thomas, 2008 [5] | Hierarchy of authority | Not reported. | Cronbach’s alpha = 0.98 | Construct (content) – expert review  Construct (structural) – confirmatory factor analysis  Construct (convergent and discriminant) – hierarchy of authority negatively correlated with participation in decision making | Health care | Multilevel | 3 |
| Okafor and Thomas, 2008 [5] | Role ambiguity | Not reported. | Cronbach’s alpha = 0.95 | Construct (content) – expert review  Construct (structural) – confirmatory factor analysis  Construct (convergent and discriminant) – role ambiguity positively associated with formalization | Health care | Multilevel | 4 |
| Sharma and Rai, 2003[6] | Hierarchy of authority | Organizational construct | Cronbach’s alpha = 0.08 | Criterion-related validity  (negative correlation with participation in decision making (r=-.03087; p<.001) | Worksite | Individual | 5 |
| **Construct: Networks and communication (3 studies, 3 measures)** | | | | | | | |
| Cloutier et al., 2009[7] | Communication | Not reported (part of “Primary Care Organizational Questionnaire”) | Cronbach’s alpha = 0.70 | Cited Hall, Tennen, Wakefield, et al, 2006  [8]; Shortell et al., 2000 [9]; Shortell et al., 1994 [10] | Health care | Organization | Not reported |
| Crea, Crampton, Knight, and Paine-Wells, 2011[11] | Team Decision Making | Not reported  (subscale of “Survey of Organizational Excellence”).  Cited Glisson, 2007 [12] | Cronbach’s alpha for overall Survey of Organizational Effectiveness = 0.97  Cronbach’s alpha for Team Decision Making = 0.91 | Cited Landuyt, 1999 [13]; Lauderdale, 1999 [14] | Community/ Public Health Services | Individual | 3 |
| Nieboer and Strating, 2012[4] | Communication | Organization’s level of internal and external communication channels. Measured with existing instruments for concepts as potential and realized capacity, connectedness and knowledge creation, and redundancy. | Cronbach’s alphas = 0.73-0.81 for subscales | Not reported | Health care | Multilevel | 6 (formal internal exchange of information); 3 (informal internal exchange of information) ; 5 (formal external exchange of information) |
| **Construct: Organizational culture (8 studies, 8 measures)** | | | | | | | |
| Caccia-Bava, Guimaraes, and Harrington, 2006[15] | Corporate culture | The pattern of values and ideas that shape human behavior in organizations. Consists of 4 types: developmental, rational, hierarchical, group | Not reported | Construct  (exploratory factor analysis) | Health care | Organization | 12  (3 per culture type) |
| Cloutier et al., 2009[7] | Conflict resolution | Not reported. Part of the “Primary Care Organizational Questionnaire (PCOQ)” | Cronbach’s alpha = 0.70 | Cited Hall et al., 2006 [8]; Shortell et al. 2006 [9]; Shortell et al., 1994 [10] | Health care | Organization | Not reported |
| Cummings, Estabrooks, Midodzi, Wallin, and Hayduk, 2007[16] | Culture | Operationalized by “freedom to make important patient care and work decisions’’ | Not reported | Criterion (predictive) | Health care | Individual | 1 |
| Lukas, Mohr, and Meterko, 2009[17] | Organizational culture | Group culture (orientation toward internal environment and processes with an emphasis on decentralization and flexibility) and hierarchical culture (orientation toward internal environment and processes but with an emphasis on centralization and control) | Cronbach’s alpha Group culture scale = 0.90; Cronbach’s Hierarchical culture scale = 0.88 | Not reported | Health care | Organization | 4 for group culture and 4 for hierarchical culture |
| Naranjo-Gil, 2009[18] | Environmental uncertainty | Organizations (e.g. managers) perceived inability to predict accurately the actions of customers and situations that comprise the external environment | Cronbach’s alpha = 0.81 | Not reported | Health care | Multilevel | 8 |
| Nieboer et al., 2011[3] | Culture | Organization’s set of values and aspirations | Cronbach’s alpha = 0.83 | Not reported | Health care | Multilevel | 2 |
| Rhodes, Lok, Yang, and Xia, 2011[19] | Organizational culture | Not reported | Cronbach’s alpha = 0.82 | Construct (structural) –confirmatory factor analysis  Criterion (Convergent/discriminant) – confirmatory factor analysis  Predictive – SEM strategic alignment associated with ERPS implementation | Business | Organization | 23 |
| Sawang and Unsworth, 2011[20] | Organizational attitude toward future innovation adoption | Attitude toward innovation adoption in the future | Cronbach’s alpha = 0.96 | Construct (structural) – confirmatory factor analysis | Worksite | Organization | Not reported |
| **Construct: Organizational climate, overall (19 studies, 32 measures)** | | | | | | | |
| **Organizational climate: Unspecified (13 studies, 23 measures)** | | | | | | | |
| Babor, Higgins-Biddle, Dauser, Higgins, and Burleson, 2005  [21] | Peer Approval for Alcohol Screening | Not reported (considered a pre-disposing factor) | Inter-rater reliability (median r = 0.70; range: v = 0.38-0.94) | Not reported | Health care | Organization | 1 |
| Babor et al., 2005  [21] | Organizational Approval for Alcohol Screening | Not reported (considered a pre-disposing factor) | Inter-rater reliability (median r = 0.70; range: v = 0.38-0.94) | Not reported | Health care | Organization | 1 |
| Beets et al., 2008[22] | School climate | Perceived administrative support and school connectedness | Cronbach’s alpha = 0.77 | Criterion (predictive) – SEM - school climate exhibited positive direct effects on teacher beliefs, teacher attitudes, and program-specific material usage (adherence) | School | Organization | 4 |
| Brink et al., 1995[23] | Characteristics of environment- social systems | Social system characteristics- Norms and support for the innovation in the social system. | Cronbach’s alpha =  0.82 (teacher)  0.75 (administrator) | Not reported | School | District | 14 (teachers);  19 (administrator) |
| Brink et al., 1995[23] | Characteristics of environment- organizational considerations | Organizational considerations- decision making latitude; looseness or tightness of authority | Cronbach’s alpha =  0.57 (teacher)  0.72 (administrator) | Not reported | School | District | 14 (teachers);  19 (administrator) |
| Aarons, Sommerfeld, and Walrath-Greene, 2009[24] | Organizational support | Processes/structures supporting the use of evidence-based practice in the organization | Kuder-Richardson 20 internal consistency = 0.81 | Construct validity – exploratory factor analysis | Health care | Organization | 9 |
| Gregory, Henry, and Schoeny, 2007[25] | School climate | Climate has been operationalized as the shared perceptions of the work environment | Cronbach’s alpha Negative Relationships = 0.75;  Administrative Leadership = 0 .96  Supportive climate = 0.93 | Construct (structural) - confirmatory factor analysis | School | Organization | 5 (Negative Relationships)  20; (Administrative Leadership)  15 (Supportive climate) |
| Livet, Courser, and Wandersman, 2008[26] | Intra-agency collaboration/  Problem solving | None provided | Cronbach’s alpha for provider agencies = 0.91;  Cronbach’s alpha for boards = 0.87 | Not reported | Health care | Organization | 12 |
| Livet et al., 2008[26] | Intra-agency communication and decision making | None provided | Cronbach’s alpha for provider agencies = 0.95;  Cronbach’s alpha for boards = 0.79 | Not reported | Health care | Organization | 10 |
| Livet et al., 2008[26] | Role clarity | None provided | Cronbach’s alpha for provider agencies = 0.96;  Cronbach’s alpha for boards = 0.77 | Not reported | Health care | Organization | 4 |
| Livet et al., 2008[26] | Shared vision | None provided | Cronbach’s alpha for provider agencies =0.93;  Cronbach’s alpha for boards=0.94 | Not reported | Health care | Organization | 4 |
| McCormick, Steckler, and McLeroy, 1995[27] | Organizational climate | Employees’ job satisfaction, their supervisors, and their involvement in decision-making. Whether organization takes appropriate risks, how conflict is managed and what motivates their work. Overall purpose was to assess the human dimension of the schools in the study | Cited Steckler, Goodman, McLeroy, Davis, and Koch, 1992 [28] | Cited Steckler et al., 1992 [28] | School | Organization | Not reported |
| Nieboer and Strating, 2012[4] | Quality improvement commitment | Degree of employee involvement in QI and human resource utilization | Cronbach’s alpha = 0.89 | Not reported | Health care | Multilevel | 14 |
| Nieboer and Strating, 2012[4] | Exploratory innovation strategy- departure | Extent to which organization departs from existing knowledge, skills, clients, markets, and products | Cronbach’s alpha = 0.86 | Not reported | Health care | Multilevel | 6 |
| Nieboer and Strating, 2012[4] | Exploitative innovation strategy- build | Extent to which organization builds on existing knowledge, skills, clients, markets and products | Cronbach’s alpha = 0.68 | Not reported | Health care | Multilevel | 6 |
| Nystrom, Ramamurthy, and Wilson, 2002[29] | Risk orientation | Attitude toward change is often a consequence and reflection of an organization’s propensity to take risk | Cronbach’s alpha = 0.84 | Construct (structural) - exploratory factor analysis  Criterion (discriminant, convergent) - joint factor analysis and scale correlations | Health care | Multilevel | 5 |
| Nystrom et al., 2002[29] | Achievement orientation | Organization’s concern for excelling | Cronbach’s alpha = 0.92 | Construct (structural) - exploratory factor analysis  Criterion (discriminant and convergent) - joint factor analysis and scale correlations | Health care | Multilevel | 2 |
| Nystrom et al., 2002[29] | External orientation | Organizations' understanding of customers' needs | Cronbach’s alpha = 0.71 | Criterion (discriminant and convergent) - joint factor analysis and scale correlations | Health care | Multilevel | 9 |
| Premkumar and Ramamurthy, 1995[30] | Internal need | Recognition of a genuine internal need within the firm to use electronic data interchange or EDI for improving its operations | Cronbach’s alpha = 0.77 | Construct (content) – pre- and pilot testing  Construct - exploratory factor analysis  Criterion - exploratory factor analysis and scale correlations | Worksite | Organization | 8 |
| Riley, Taylor, and Elliott, 2001[31] | Capacity | Skills and resources of an organization to undertake program activities. | Cronbach’s alpha = ranged 0.84-0.92 | Construct (content) - expert review | Community/  public health/social services | Organization | 18 |
| Sawang and Unsworth, 2011[20] | Implementation climate | Perception of managerial expectations of the extent to which employees supported the implementation of the innovation | Cronbach’s alpha = 0.92 | Construct (structural) – confirmatory factor analysis | Worksite | Organization | 3 |
| Sawang and Unsworth, 2011[20] | Implementation policies and practices | Extent to which organization endorsed policies and practices such as training, rewards or incentives, innovation assistance, time for participating in innovation implementation, and communication about innovation implementation | Cronbach’s alpha = 0.081 | Construct (structural) – confirmatory factor analysis | Worksite | Organization | 6 |
| Steckler, Goodman, 1992[28] | Organizational climate | Not reported | Cronbach’s alpha = 0.92 | Construct | School | Organization | 32 |
| **Organizational climate: Learning climate (3 studies, 3 measures)** | | | | | | | |
| Choi and Chang, 2009[32] | Support for learning | The extent to which an agency encourages and provides a supportive environment for learning-related activities | Cronbach’s alpha = 0.84 | Not reported | Community/  public health/social services | Organization | 4 |
| Lin, 2008[33] | Organizational learning | Organizational learning refers to the capacity or processes within a firm enabling the acquisition of, access to and revision of organizational memory, thereby providing directions for organizational action  Components include:   - Managerial commitment - Systems orientation - Knowledge acquisition | Internal consistency reliability;  Composite reliability scores ranged from 0.82- 0.93 for the four components of the scale;=0.93 for managerial commitment; Composite reliability score= 0.82 for systems orientation; Composite reliability score= 0.90 for knowledge acquisition ; Composite reliability score= 0.88 for knowledge dissemination | Construct (structural) - confirmatory factor analysis  Convergent - loading for all items exceeds the recommended level of 0.7, and all factor loadings are statistically significant at p .0001 | Worksite | Organization | 4 (managerial commitment); 4 (systems orientation); 8 (knowledge acquisition) |
| McGowan and Madey, 1998[34] | Organizational learning | Comprised of 2 factors: (1) Training availability, and (2) Level of technical expertise  Training availability refers to the amount of education that is made available to the implementers of technology.  Level of technical expertise refers to the level of specialized knowledge and technical expertise in an organization for implementing an electronic data interchange or EDI | Cronbach’s alpha = 0.81 for training availability;  Cronbach’s alpha = =0.75 for level of technical knowledge | Criterion (predictive) | Businesses | Organization | 2 (training availability); 4 (level of technical expertise and EDI knowledge) |
| **Organizational climate: Compatibility (3 studies, 3 measures)** | | | | | | | |
| Rahimi, Timpka, Vimarlund, Uppugunduri, & Svensson, 2009[35] | Compatibility | The degree to which an innovation is consistent with existing values and needs of users | Differences in respondent profile and level of agreement were tested for significance using chi-square test (and Fisher's Exact Test when necessary) | Face validity  Construct (content) – pilot testing | Health care | Organization | Not reported |
| Brink et al., 1995[23] | Compatibility | The compatibility of the innovation with existing practices | Cronbach’s alpha =0.78 for teachers; Cronbach’s alpha =  0.66 for administrators | Criterion –dissemination strategies did not increase teachers or administrators’ receptivity toward the innovation. In these analyses, compatibility was an aspect of receptivity to the innovation | School | Organization | 6 (teachers);  5 (administrators) |
| Premkumar and Ramamurthy, 1995[30] | Organizational compatibility | Perception of electronic data interchange or EDI technology to be compatible with existing operating practices, and beliefs and value systems | Cronbach’s alpha = 0.82  Cited Cooke, et al, 1998 [2] | Construct (content) - pre and pilot testing; Construct - exploratory factor analysis; Criterion (convergent and discriminant) -principal component factor analysis | Worksite | Organization | 2 |
| **Organizational climate: Goals and feedback (2 studies, 3 measures)** | | | | | | | |
| Cooke, 2000  [1] | Clarity/work climate | How explicitly rules and policies are communicated | Cronbach’s alpha  > 0.70  Cited Cooke, et al, 1998 [2] | Not reported | Health care | Organization | 9 |
| Cooke, 2000  [1] | Innovation | The emphasis on variety change and new approaches | Cronbach’s alpha  > 0.70  Cited Cooke, et al, 1998 [2] | Not reported | Health care | Organization | 9 |
| Nieboer et al., 2011[3] | Strategy | The activities in response to or in anticipation of changes in the external environment (customers, competitors, and government) | Cronbach’s alpha = 0.82 | Not Reported | Health care | Organization | 3 |
| **Construct: Readiness for implementation, overall (21 studies, 28 measures)** | | | | | | | |
| **Readiness for implementation, unspecified (3 studies, 6 measures)** | | | | | | | |
| Asgary-Eden and Lee, 2012[36] | Organizational readiness for change (ORC)  Cited Lehman, Greener, and Simpson, 2002 [37] | Motivation and personality attributes of program leaders and staff, institutional resources, and organizational climate | Cronbach’s alpha for Office Resources subscale = 0.74-0.84; Training needs subscale = 0.83-.86 | Cited Lehman et al. 2002 [37] | Community/  public health/social services | Organization | 4 (office resources); 8 (training needs) |
| Krumwiede, 1998[38] | Organizational factors | Level of top management support | Cronbach’s alpha = 0.68 | Not reported | Worksite | Organization | 3 |
| Krumwiede, 1998[38] | Organizational factors | Level of non-accounting ownership | Cronbach’s alpha = 0.72 | Not reported | Worksite | Organization | Not reported |
| Krumwiede, 1998[38] | Organizational factors | Level of clarity and consensus about innovation objectives | Cronbach’s alpha = 0.72 | Not reported | Worksite | Organization | 2 |
| Krumwiede, 1998[38] | Organizational factors | Level of training provided | Cronbach’s alpha = 0.91 | Not reported | Worksite | Organization | 3 |
| Riley et al., 2001[31] | Organizational predisposition | Motivation to undertake health promotion activities. Operationally defined as collective belief among staff in importance of conducting these activities | Cronbach’s alpha = 0.61-0.87 | Construct (content) – expert review  Criterion – predictive and concurrent | Community/  Public Health/Social Services | Organization | 18 |
| **Readiness for implementation: Leadership engagement (17 studies, 17 measures)** | | | | | | | |
| Becan, Knight, and Flynn, 2012 [39] | Leadership | Staff perception of program director behavior | Cronbach’s alpha =0.92 | Criterion (predictive) – regression analysis – positive perceptions of leadership practices positively associated with individual measures of innovation adoption; Criterion (convergent) - the program leadership scale has shown good convergent validity with measures of job satisfaction and burnout  Cited Broome, Knight, Edwards, and Flynn, 2009 [40] | Health care | Organization | 9 |
| Birken, Lee, Weiner, Chin, and Schaefer, 2013  [41] | Middle managers’ commitment | Middle managers’ commitment to innovation implementation is a behavioral manifestation of their emotional attachment to, identification with, and involvement in innovation implementation | Cronbach’s alpha = 0.75 | Criterion (predictive) regression – middle manager commitment to innovation implementation positively associated with linkages with community resources (an aspect of implementation). Not significantly associated with two other aspects of implementation – promoting patient self-management or delivery system redesign. | Health care | Organization | 3 |
| Caccia-Bava et al., 2006[15] | Strategic leadership | The ability of the top management team to provide leadership when the organizational environment requires change: both transactional and charismatic leadership were measured | Cronbach’s alpha = 0.88 for transactional leadership  Cronbach’s alpha = 0.81 for charismatic leadership: | Construct (content) – expert review; Construct (structural) – confirmatory factor analysis; Criterion (predictive) – strategic leadership (combined transactional and charismatic) associated with effectiveness of implementing change | Health care | Organization | 5 (transactional leadership); 7 (charismatic leadership) |
| Choi and Chang, 2009[32] | Management support | Level of management support for the innovation | Cronbach’s alpha = 0.90 | Not reported | Community/public health/social services | Organization | 4 |
| Cloutier et al., 2009[7] | Leadership | Measured as part of the “Primary Care Organizational Questionnaire (PCOQ)” | Cronbach’s alpha = 0.70  Cited Hall et al., 2006  [8]; Shortell et al., 2000 [9]; and Shortell et al., 1994 [10] | Cited Hall et al., 2006,  [8]; Shortell et al., 2000 [9]; and Shortell et al., 1994 [10] | Health care | Organization | Not reported |
| Cooke, 2000  [1] | Supervisor support | The degree to which management are supportive of staff | Cronbach’s alpha = 0.70  Cited Cooke et al., 1998 [2] | Criterion (predictive)  Cited Cooke et al., 1998 [2] | Health care | Organization | 9 |
| Cummings et al., 2007[16] | Leadership | A nurse manager or immediate supervisor who is a good leader or manager | Not reported | Better context (positive culture, good leadership, positive evaluation) positively associated with more research utilization | Health care | Individual | 1 |
| Damanpour and Schneider, 2006  [42] | Top managers’ attitude | Dimensions of top managers’ attitude toward new public management programs… clear, transformational leadership that supports teamwork and staff involvement in decision making | Cronbach’s alpha: Traditional= 0.24; Favoring Competition = 0.63; Entrepreneurial = 0.59 | Construct validity(structural) – exploratory factor analysis; Criterion related validity (predictive) – favoring competition and entrepreneurial associated with innovation initiation, adoption, and implementation | Community/public health/social services | Organization | 10 |
| Gregory et al., 2007[25] | Administrative leadership (part of climate construct) | Climate has been operationalized as the shared perceptions of the work environment | Cronbach’s alpha: Negative Relationships = 0.75; Administrative Leadership = 0.96; Supportive climate = 0.93 | Construct (structural) - confirmatory factor analysis | School | Organization | 5 (Negative Relationships); 20(Administrative Leadership); 15 (supportive climate) |
| Law and Ngai, 2007  [43] | Senior management support | How supportive senior management is towards IT initiatives | A Cronbach’s alpha > 0.70 | Not reported | Worksite | Organization | 2 |
| Livet et al., 2008[26] | Leadership | Ability to foster respect, resolve conflicts, combine different perspectives and opinions, use resources appropriately, and empower Staff’ | Cronbach’s alpha = 0.88; Cronbach’s alpha = 0.73 | Not reported | Health care | Organization | 10 |
| Lukas et al., 2009[17] | Management support | The presence of practical expressions of management support for the Advanced Clinic Access initiative at the facility | Personal leadership support: Cronbach’s alpha = 0.89; Practical management support: Not reported | Not reported | Health care | Multilevel (personal and facility) | Personal leadership support: (7 item scale); Practical (facility) management support (8 dichotomous items) |
| Nieboer et al., 2011[3] | Management style | Symbolic (exemplary) behavior such as managerial time spending patterns and abilities to grasp opportunities to realize change | Cronbach’s alpha = 0.80 | Cited Jamrog et al., 2006 [44]. | Health care | Organization | 4 |
| Okafor and Thomas, 2008 [5] | Openness to change | Organizational leaders’ attitude to change influences the organizations innovativeness | Cronbach's alpha = 0.92 | Construct (content) – expert review; Construct (Structural) – confirmatory factor analysis; Construct (convergent and discriminant) – extraversion positively correlated with openness to change; Criterion (Predictive) – regression – openness to change positively associated with innovation adoption-enabling elements | Health care | Multilevel | 5 |
| Premkumar and Ramamurthy, 1995[30] | Top management support | Support assessed by electronic data interchange or EDI-related use, risk-taking, commitment to provide finances and other resources, and, vision for leadership role. | Cronbach’s alpha = 0.89 | Construct (content) - pre and pilot-testing; Construct (structural) – exploratory factor analysis; Criterion (convergent and discriminant) – exploratory factor analysis and scale correlations | Worksite | Organizational | 3 |
| Rhodes et al., 2011[19] | Strategic alignment/ leadership commitment | Not reported | Cronbach’s alpha = 0.85 | Construct validity (structural) – exploratory factor analysis; Criterion related validity (convergent and discriminant) – exploratory factor analysis and scale correlations | Business | Organization | 3 |
| Sawang and Unsworth, 2011[20] | Top management support | Extent to which top management supports and is committed to the implementation process. | Cronbach’s alpha = 0.77 | Construct validity (structural) – confirmatory factor analysis | Worksite | Organization | 3 |
| **Readiness for implementation: Available resources (4 studies, 5 measures)** | | | | | | | |
| Choi and Chang, 2009[32] | Resource availability | The extent to which an agency provides resources for innovation | Cronbach’s alpha = 0.78 | Not reported | Community/public health/social services | Organization | 4 |
| Nystrom et al., 2002[29] | Organization slack | “Slack” resources provide an organization with a cushion of spare resources that prevents it from fatal hazards in the face of a rapidly changing environment. | Cronbach’s alpha = 0.61 | Criterion validity | Health care | Multilevel | 4 |
| Premkumar and Ramamurthy, 1995[30] | Information system infrastructure | Telecommunications and database infrastructure | Cronbach’s alpha = 0.71 | Construct (content) – pre and pilot-testing; Construct (structural) – exploratory factor analysis; Criterion validity (convergent and discriminant) – exploratory factor analysis and scale correlations | Worksite | Organizational | 3 |
| Sawang and Unsworth, 2011[20] | Financial resources availability | Financial resource allocation within the company | Cronbach’s alpha = 0.76 | Construct validity (structural) – confirmatory factor analysis | Worksite | Organization | 3 |
| Sawang and Unsworth, 2011[20] | Human resources availability | Availability of skilled labor and managerial talent | Cronbach’s alpha = 0.73 | Construct validity (structural) – confirmatory factor analysis | Worksite | Organization | 2 |

**REFERENCES:**

1. Cooke M: **The dissemination of a smoking cessation program: predictors of program awareness, adoption and maintenance.** *Health Promotion International* 2000, **15:**113-124.

2. Cooke M, Mattick RP, Campbell E: **The influence of individual and organizational factors on the reported smoking intervention practices of staff in 20 antenatal clinics.** *Drug and alcohol review* 1998, **17:**175-185.

3. Nieboer AP, Pijpers V, Strating MH: **Implementing Community Care for People with Intellectual Disability: The Role of Organizational Characteristics and the Innovation’s Attributes.** *Journal of Applied Research in Intellectual Disabilities* 2011, **24:**370-380.

4. Nieboer AP, Strating MM: **Innovative culture in long-term care settings: the influence of organizational characteristics.** *Health Care Manage Rev* 2012, **37:**165-174.

5. Okafor MC, Thomas J: **Presence of Innovation Adoption-Facilitating Elements in Hospitals, and Relationship to Implementation of Clinical Guidelines.** *Annals of Pharmacotherapy* 2008, **42:**354-360.

6. Sharma S, Rai A: **An assessment of the relationship between ISD leadership characteristics and IS innovation adoption in organizations.** *Information & Management* 2003, **40:**391-401.

7. Cloutier MM, Wakefield DB, Tsimikas J, Hall CB, Tennen H, Brazil K: **Organizational attributes of practices successful at a disease management program.** *J Pediatr* 2009, **154:**290-295.

8. Hall CB, Tennen H, Wakefield DB, Brazil K, Cloutier MM: **Organizational assessment in paediatric primary care: development and initial validation of the primary care organizational questionnaire.** *Health Services Management Research* 2006, **19:**207-214.

9. Shortell SM, Jones RH, Rademaker AW, Gillies RR, Dranove DS, Hughes EF, Budetti PP, Reynolds KS, Huang C-F: **Assessing the impact of total quality management and organizational culture on multiple outcomes of care for coronary artery bypass graft surgery patients.** *Medical care* 2000, **38:**207-217.

10. Shortell SM, Zimmerman JE, Rousseau DM, Gillies RR, Wagner DP, Draper EA, Knaus WA, Duffy J: **The performance of intensive care units: does good management make a difference?** *Medical care* 1994**:**508-525.

11. Crea TM, Crampton DS, Knight N, Paine-Wells L: **Organizational factors and the implementation of family to family: contextual elements of systems reform.** *Child Welfare* 2011, **90:**143-161.

12. Glisson C: **Assessing and Changing Organizational Culture and Climate for Effective Services.** *Research on Social Work Practice* 2007, **17:**736-747.

13. Landuyt NG: *Employee perceptions of organizational quality and learned helplessness in higher education.* The University of Texas at Austin; 1999.

14. Lauderdale M: *Reinventing texas government.* University of Texas Press; 2010.

15. Caccia-Bava MC, Guimaraes T, Harrington SJ: **Hospital organization culture, capacity to innovate and success in technology adoption.** *J Health Organ Manag* 2006, **20:**194-217.

16. Cummings GG, Estabrooks CA, Midodzi WK, Wallin L, Hayduk L: **Influence of organizational characteristics and context on research utilization.** *Nurs Res* 2007, **56:**S24-39.

17. Lukas CV, Mohr DC, Meterko M: **Team effectiveness and organizational context in the implementation of a clinical innovation.** *Qual Manag Health Care* 2009, **18:**25-39.

18. Naranjo-Gil D: **The influence of environmental and organizational factors on innovation adoptions: Consequences for performance in public sector organizations.** *Technovation* 2009, **29:**810-818.

19. Rhodes J, Lok P, Yang S, Xia Y: **The effects of organizational intangible factors on successful enterprise resource planning systems implementation and organizational performance: A China experience.** *Asian Business & Management* 2011, **10:**287-317.

20. Sawang S, Unsworth KL: **A model of organizational innovation implementation effectiveness in small to medium firms.** *International Journal of Innovation Management* 2011, **15:**989-1011.

21. Babor TE, Higgins-Biddle J, Dauser D, Higgins P, Burleson JA: **Alcohol screening and brief intervention in primary care settings: implementation models and predictors.** *J Stud Alcohol* 2005, **66:**361-368.

22. Beets MW, Flay BR, Vuchinich S, Acock AC, Li KK, Allred C: **School climate and teachers' beliefs and attitudes associated with implementation of the positive action program: a diffusion of innovations model.** *Prev Sci* 2008, **9:**264-275.

23. Brink SG, Basen-Engquist KM, O'Hara-Tompkins NM, Parcel GS, Gottlieb NH, Lovato CY: **Diffusion of an effective tobacco prevention program. Part I: evaluation of the dissemination phase.** *Health Education Research* 1995, **10:**283-295.

24. Aarons G, Sommerfeld D, Walrath-Greene C: **Evidence-based practice implementation: The impact of public versus private sector organization type on organizational support, provider attitudes, and adoption of evidence-based practice.** *Implementation Science* 2009, **4:**83.

25. Gregory A, Henry DB, Schoeny ME: **School climate and implementation of a preventive intervention.** *Am J Community Psychol* 2007, **40:**250-260.

26. Livet M, Courser M, Wandersman A: **The prevention delivery system: organizational context and use of comprehensive programming frameworks.** *Am J Community Psychol* 2008, **41:**361-378.

27. McCormick L, Steckler A, McLeroy KR: **Diffusion of Innovations in Schools: A Study of Adoption and Implementation of School-Based Tobacco Prevention Curricula.** *American Journal of Health Promotion* 1995, **9:**210-219.

28. Steckler A, Goodman RM, McLeroy KR, Davis S, Koch G: **Measuring the diffusion of innovative health promotion programs.** *Am J Health Promot* 1992, **6:**214-224.

29. Nystrom PC, Ramamurthy K, Wilson AL: **Organizational context, climate and innovativeness: adoption of imaging technology.** *Journal of Engineering and Technology Management* 2002, **19:**221-247.

30. Premkumar G, Ramamurthy K: **The Role of Interorganizational and Organizational Factors on the Decision Mode for Adoption of Interorganizational Systems*.** *Decision Sciences* 1995, **26:**303-336.

31. Riley BL, Taylor SM, Elliott SJ: **Determinants of implementing heart health: promotion activities in Ontario public health units: a social ecological perspective.** *Health Educ Res* 2001, **16:**425-441.

32. Choi JN, Chang JY: **Innovation implementation in the public sector: an integration of institutional and collective dynamics.** *J Appl Psychol* 2009, **94:**245-253.

33. Lin H: **Empirically testing innovation characteristics and organizational learning capabilities in e‐business implementation success.** *Internet Research* 2008, **18:**60-78.

34. McGowan MK, Madey GR: **The influence of organization structure and organizational learning factors on the extent of EDI implementation in U.S. firms.** *Inf Resour Manage J* 1998, **11:**17-27.

35. Rahimi B, Timpka T, Vimarlund V, Uppugunduri S, Svensson M: **Organization-wide adoption of computerized provider order entry systems: a study based on diffusion of innovations theory.** *BMC Medical Informatics and Decision Making* 2009, **9:**52.

36. Asgary-Eden V, Lee CM: **Implementing an evidence-based parenting program in community agencies: what helps and what gets in the way?** *Adm Policy Ment Health* 2012, **39:**478-488.

37. Lehman WE, Greener JM, Simpson DD: **Assessing organizational readiness for change.** *J Subst Abuse Treat* 2002, **22:**197-209.

38. Krumwiede KR: **The implementation stages of activity-based costing and the impact of contextual and organizational factors.** *Journal of Management Accounting Research* 1998, **10:**239.

39. Becan JE, Knight DK, Flynn PM: **Innovation adoption as facilitated by a change-oriented workplace.** *Journal of substance abuse treatment* 2012, **42:**179-190.

40. Broome KM, Knight DK, Edwards JR, Flynn PM: **Leadership, burnout, and job satisfaction in outpatient drug-free treatment programs.** *Journal of Substance Abuse Treatment* 2009, **37:**160-170.

41. Birken SA, Lee S-YD, Weiner BJ, Chin MH, Schaefer CT: **Improving the effectiveness of health care innovation implementation middle managers as change agents.** *Medical Care Research and Review* 2013, **70:**29-45.

42. Damanpour F, Schneider M: **Phases of the Adoption of Innovation in Organizations: Effects of Environment, Organization and Top Managers1.** *British Journal of Management* 2006, **17:**215-236.

43. Law CC, Ngai EW: **ERP systems adoption: An exploratory study of the organizational factors and impacts of ERP success.** *Information & Management* 2007, **44:**418-432.

44. Jamrog J., Bear D. J., Chaves W. V., Conte S. DDJ, Hipple J. LMB, Risenberg R., Taylor G., R. V: *The quest for innovation: a global study of innovation management 2006-2016.* American Management Association; 2006.
